# The Ear in Subterranean Rodents Revisited: Cochlear Hair‐Cell Populations in African Mole‐Rats (Bathyergidae)

**DOI:** 10.1002/jmor.70106

**Published:** 2025-12-17

**Authors:** Lucie Svačinová, Simone Lange, Matěj Lövy, Barbora Konopová, Nigel Charles Bennett, Daniel William Hart, Radim Šumbera, Hynek Burda

**Affiliations:** ^1^ Department of Zoology, Faculty of Science University of South Bohemia České Budějovice Czech Republic; ^2^ Department of General Zoology, Faculty of Biology University Duisburg‐Essen Essen Germany; ^3^ Institute of Entomology Biology Centre of the Czech Academy of Sciences České Budějovice Czech Republic; ^4^ Mammal Research Institute, Department of Zoology and Entomology University of Pretoria Pretoria South Africa; ^5^ Department of Game Management and Wildlife Biology, Faculty of Forestry and Wood Sciences Czech University of Life Sciences Praha Czech Republic

**Keywords:** bathyergidae, cochlea, ear morphology, hearing, subterranean mammals

## Abstract

Based on von Békésy's premise that “The physical laws served as guidelines for the evolution of the structures and functions of the middle and inner ear,” we aimed to understand how the unique subterranean acoustic environment, which promotes the propagation of low‐frequency sounds and thereby selects for enhanced low‐frequency hearing, influences functional adaptations reflected in the morphological convergence of the cochlea in subterranean African mole‐rats (Bathyergidae). We conducted a morphometric analysis of the cochlea in 12 species representing all six genera of African mole‐rats, spanning a body mass range of 30–2000 g. Cochlear partitions were examined using light microscopy following the standard surface specimen technique. The mole‐rat cochleae has 3–4.3 coils. The length of the basilar membrane (BM) varies from 6.5 to 15.6 mm. Mean densities of inner hair cells (IHC) range from 104 to 122, whereas outer hair cells (OHC) range from 390 to 480 per 1 mm. Hair cell density increased slightly from the base towards the apex in all species studied. The radial width of the cuticular plates of the three rows (triad) of OHC, shown in previous studies to mirror BM width, increased continuously from, on average, 22 ± 3 µm at the base to 35 ± 6 μm at the apex. Length of BM, width of the OHC triad and total number of hair cells (and thus hearing resolution capabilities) are related to body size. When compared to other mammals, the cochleae of bathyergids exhibit quantitative characteristics that closely resemble the apical regions of the cochleae in other species—specifically, those segments tuned to low frequencies. Moreover, the width of OHC triads was strongly correlated with the tonotopic organization of frequencies along the organ of Corti, confirming its value as a structural predictor of auditory capability.

## Graphical abstract

Morphometric analysis of the cochlea was conducted on 12 species of African mole‐rats (Bathyergidae), revealing that cochlear features related to hearing resolution correlate with body size. Compared to other mammals, bathyergid cochleae resemble the apical, low‐frequency‐tuned regions of typical mammalian cochleae. A correlation was found between the width of the outer hair cell (OHC) triad and tonotopic frequency mapping in the organ of Corti.

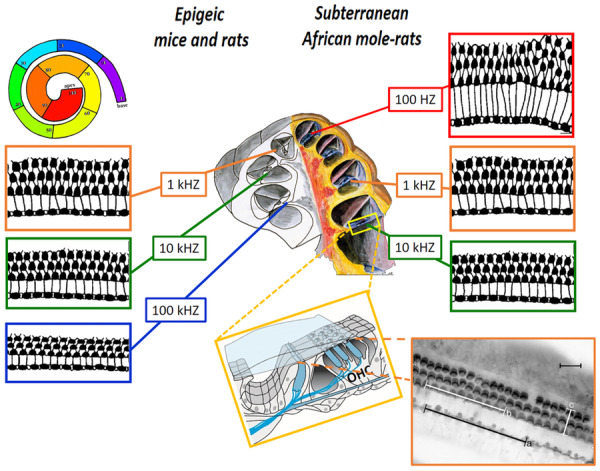

## 1 Introduction

Across the globe, more than 5% of mammalian species, representing several orders, spend most of their lives in moist and dark, oxygen‐poor and carbon dioxide‐rich, self‐constructed underground burrow systems that are deprived of most sensory cues available aboveground. These mammals are specialized for their unique way of life, conducting all essential activities, including foraging, mating, breeding and sleeping, underground. They thus represent one of the most striking examples of convergent evolution across multiple levels of biological organization (Nevo 1999).

The dark, monotonous environment of burrows lacks orientation cues that are readily available to animals living aboveground, and almost all communication channels, including the acoustic one, are dramatically constrained (reviewed in Begall, Burda, et al. 2007; Burda et al. 1990; Lacey 2000; Nevo 1999). Airborne sound transmission in tunnels is limited due to a quick attenuation and masking by high levels of background noise. Frequencies between 200 and 800 Hz are best propagated in this niche (Heth et al. 1986; Lange et al. 2007). Interestingly, these low‐frequency sounds are not only the least attenuated, but they can even be amplified due to the so‐called stethoscope effect (Lange et al. 2007; Schleich and Antenucci 2009).

Vocalization and hearing in subterranean mammals are well adapted to the burrow acoustics. The main frequencies used in communication usually range between 0.5 and 4 kHz, and the best hearing sensitivity is between 1 and 2 kHz (reviewed in Begall, Lange, et al. 2007; Burda 2006; Schleich et al. 2007). Their hearing range usually starts below 1 kHz and reaches only 6−12 kHz, hearing sensitivity is consequently low, with the lowest threshold typically at 30 dB SPL (Brückmann and Burda 1997; Caspar et al. 2021; Gerhardt et al. 2017; Heffner and Heffner 1990, 1992, 1993; Kössl et al. 1996; Müller and Burda 1989; Müller et al. 1992; Pyott et al. 2020).

Low‐frequency (< 2 kHz) sounds are characterized by longer (> 17 cm) waves, which cannot be easily localized by small and medium‐sized mammals (with a smaller head size, and thus shorter interaural distance) and hearing and vocalization in those mammals is thus typically tuned to higher frequencies (reviewed in Heffner and Heffner 2016). Unique low‐frequency hearing of subterranean mammals, which are all small mammals, used to be denoted either as degenerated (e.g., Heffner and Heffner 1993) or as specialized and adaptive (e.g., Burda 2006). It is associated with several morphological peculiarities of the outer and middle ear such as reduction of the pinnae, enlarged incus resulting in a small malleus‐to‐incus lever ratio, and an enlarged stapedial footplate resulting in a small eardrum‐to‐stapedial footplate area ratio (Begall, Lange, et al. 2007; Burda et al. 1992; Mason 2001, 2013). The cochlea of the inner ear in all the placental mammals studied thus far (and subterranean mammals are not exceptional in this respect) exhibits the same common morphological plan and organization. However, in subterranean mammals, the cochlea exhibits a tower‐like structure with a relatively (compared to epigeic relatives) higher number of coils (Begall, Lange, et al. 2007; Müller et al. 1989). The cochlear partition (the basilar membrane [BM] carrying the organ of Corti) plays a major role in the cochlear mechanics affecting the hearing capabilities of mammals. The organ of Corti in placental mammals is organized into a remarkable geometric pattern consisting of three parallel rows of outer hair cells (OHC) and a single parallel row of inner hair cells (IHC), separated by a tunnel of Corti. The upper surface of the Corti organ, as seen on the surface specimens (cf. Methods), is formed by the reticular lamina composed of cuticular plates of cells. Cuticular plates of hair cells carry sensory hairs, stereocilia.

The cochlea in mammals is organized tonotopically, which means that each frequency is registered within a specific segment of BM, with short waves of high‐frequency sound causing maximum displacement of the BM in its basal part, whereas longer waves of low frequencies propagate further from the oval window and cause maximum displacement of the BM at its apical part (von Békésy 1960). The difference in the wave travelling along the cochlear duct is facilitated by a stiffness gradient of BM, which depends on its width and thickness (von Békésy 1960). In mammals examined to date, the width of the BM rises from the base to the apex, while its thickness decreases.

According to von Békésy (1960), the width and thickness of the BM are two primary parameters determining the stiffness of the BM. The basoapical change of stiffness along the BM results in basoapical frequency distribution, tonotopy. The method of preparation for the surface specimens of the cochlear partition used here does not enable us to measure BM thickness and BM width accurately. Instead, we focused on the width of the OHC triad (actually the radial width of the cuticular plates of the three rows of OHC on the reticular lamina of the organ of Corti), which can be precisely measured on surface specimens of the cochlear partition (Burda 1985). This parameter follows and correlates (with a factor × 5.2–6.1) with the basoapical changes of BM width along the length of the cochlear spiral (Burda et al. 1988). Moreover, it reflects the ontogenic maturation of the organ of Corti (Burda 1985; Burda and Branis 1988).

In most mammalian species studied to date, the distribution of the frequencies of the hearing range along the BM is more or less regular, that is, a BM segment of approximately the same length processes each octave of the hearing range (Greenwood 1990; Liberman 1982; Müller 1991, 1996). However, in mammals highly specialized for perception of certain frequencies, such as in some echolocating bats, the octave of the best hearing can be processed along a longer part of the BM than other octaves of the hearing range (Bruns and Schmieszek 1980; Russell and Kössl 1999; Vater et al. 1985). This phenomenon is called the “acoustic fovea” in analogy with the retinal fovea (Schuller and Pollak 1979). Among subterranean mammals, the acoustic fovea was first described in Ansell's mole‐rat (*Fukomys anselli*) (Müller et al. 1992), with additional evidence suggesting its presence in the blind mole‐rat (*Nanospalax ehrenbergi*) and the Gansu zokor (*Eospalax cansus*) (Bruns et al. 1988; Pleštilová et al. 2019, respectively).

The African mole‐rats (Bathyergidae) have become the most widely and deeply studied group of subterranean mammals in terms of acoustic communication, hearing, and otomorphological adaptations to the specific acoustic environment (specific references above and below). The various members of the family Bathyergidae share a similar morphology at first glance, differing, in their body size (ranging from 30 g in *Heterocephalus glaber* to 2 kg in *Bathyergus suillus*), colouration, pelage development (ranging from “hairless” naked mole‐rat, *H. glaber*, to densely furry silvery mole‐rat, *Heliophobius argenteocinereus*), social organisation (comprising genera with solitary as well as highly social lifestyles), and ecological and geographic distribution (Bennett and Faulkes 2000; Burda et al. 2018). While *Bathyergus* excavates tunnels mainly by scratching using their forefeet, the other five genera use procumbent incisors to loosen the soil (Bennett and Faulkes 2000; Gomes Rodrigues et al. 2023). African mole‐rats are strictly subterranean, meaning that they forage underground. The largest bathyergid, the Cape dune mole‐rat (*B. suillus*), however, appears to frequently move above‐ground (Bennett et al. 2009; Bray et al. 2012).

**Figure 1:**
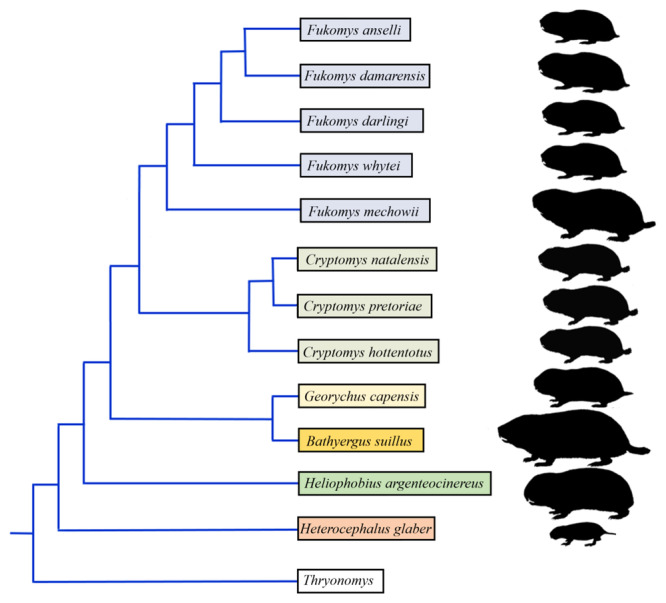
Cladogram illustrating the phylogenetic relationships among bathyergid genera and species (Šumbera et al. 2023, 2024).

Given the close phylogenetic relationship (Figure 1), uniform morphotype, and common subterranean ecology of bathyergids, yet differences in body size, the question thus arises as to what extend these differences are reflected in hearing. It is a generally accepted fact that larger vertebrates have a hearing range shifted to lower frequencies than their smaller relatives living in the same acoustic environment (reviewed in Dusenbery 1992; Heffner and Heffner 2016). On the other hand, burrowing rodents with regular bouts of surface activity exhibit differences in hearing range and sensitivity compared to strictly underground dwellers, as reported for coruros (*Spalacopus cyanus*) (Begall et al. 2004) and prairie dogs (*Cynomys* sp.) (Heffner et al. 1994). Although hearing has not been studied in *Bathyergus* mole‐rats, we may assess its features from the ear morphology. As von Békésy (1974) proclaimed: “The physical laws served as guidelines for the evolution of the structures and functions of the middle and inner ear.” Indeed, ear morphology may reveal how and what mammals hear and also where and how they live (e.g., Hemilä et al. 1995; Webster and Webster 1975). Comparative morphological study of the ear has great potential for functional interpretations.

While the middle ear of bathyergids has been described comprehensively and in detail (Burda et al. 1992; Fleischer 1973; Lange 2005; Mason et al. 2016), information on the cochlea of the inner ear remains fragmentary (Burda et al. 1988; Fleischer 1973; Mason et al. 2016; Müller et al. 1989, 1992; Peacock et al. 2025). The organ of Corti has been studied in detail in three *Fukomys* species and in three subspecies of *Cryptomys hottentotus* (Lange 2005; Müller et al. 1992), some specific aspects of hair cell innervation in *H. glaber* and *Fukomys damarensis* (Barone et al. 2019), yet comparative data for the other bathyergid genera are missing.

Accordingly, we aimed to expand the comparative dataset by examining the cochleae of representatives from all African mole‐rat genera, encompassing the full range of body sizes and ecological lifestyles within this family, with the goal of improving understanding of cochlear functional adaptations to a mammal life history.

## 2 Materials and Methods

We examined 66 cochleae of 52 individuals representing 12 species encompassing all six bathyergid genera. All animals were healthy adults, both sexes were approximately equally represented, average body mass and condylobasal length (CBL) of the skull are given in Table 1. The animals examined in the present study were either sacrificed in the framework of other studies (Kverková et al. 2018; Lange 2005; Patzenhauerová et al. 2010) or collected in cooperation with the University of Pretoria. The specimens are stored at the Faculty of Sciences, University of South Bohemia, under the accession numbers given in the Table 1.

**Table 1:** The list of studied African mole‐rat species.

Species	*N* individuals	*N* cochleae	Locality	CBL (mm)	Body mass^a^ (g)	CBL^a^ (mm)	Accesion numbers of specimens
*Bathyergus suillus*	6	6	RSA, Darling	57.2 ± 8.1	783	60.1	BS2‐BS4, BS6‐BS8
*Cryptomys hottentotus*	3	4	RSA, Kamieskroon, Darling	34.1	56	30.6	Lange (2005)
	6	6		36.1 ± 1.9			CHH1‐CHH6,
*Cryptomys natalensis*	3	5	RSA, Glengarry	37.6	97	37.6	Lange (2005)
*Cryptomys pretoriae*	3	6	RSA, Pretoria	38.2	84	37.9	Lange (2005)
*Fukomys anselli*	3	4	Zambia, Lusaka	33.1	88	32.8	Lange (2005)
*Fukomys damarensis*	3	5	Namibia, Dordabis	39.2	140	35.7	Lange (2005)
*Fukomys darlingi*	5	10	Zimbabwe, Goromonzi	36.2	77	32.9	Lange (2005)
*Fukomys mechowii*	4	4	Zambia, Ndola^b^	49.8 ± 2.2	307	47.1	9330, 9655, 9658, 8280
*Fukomys whytei*	4	4	Tanzania, Kalolondwe	36.9 ± 1.7	127	37.5	472, 473, 477, 478
*Georychus capensis*	4	4	RSA, Darling	39.4 ± 2.0	180	46.7	GC1‐GC4
*Heliophobius argenteocinereus*	4	4	Tanzania, Nayala	48.0 ± 4.0	176	38.6	382–384, 394
*Heterocephalus glaber*	4	4	ZOO Dresden, ZOO Munster^b^	25.5 ± 0.5	34	21.4	2075, 1528, 7146, 2509

*Note:* Standard deviations (SDs) for CBL in several species could not be reported, as the original dataset from Lange (2005) is no longer accessible.

^a^ Values of mean body mass and mean condylobasal length of the skull (CBL) were taken from Happold (2013), Bennett and Faulkes (2000) and Burda et al. (2005);

^b^ Examined individuals were born in captivity.

### 2.1 Specimen Preparation

The animals were either perfused with heparinized PBS (pH 7.4) followed by 4% buffered paraformaldehyde (PFA) and stored for several days in 0.5% PFA, or immediately after death (by overdose of chloroform) decapitated, the heads were skinned, and 10% solution of formaldehyde was injected into the ear canal and its surrounding. The heads were then stored in 10% formaldehyde and subsequently in 0.5% formaldehyde or for several weeks in 1% buffered PFA and subsequently in 0.5% buffered PFA.

Left (or both) bullae were extracted and opened from the lateral side. The eardrum and middle ear ossicles were removed, and the cochlea was dissected turn by turn, starting from the apex with the standard surface specimen technique (Burda 1985). The bony shell was removed, and the cochlear duct was stained with toluidine blue and Ehrlich's hematoxylin. The cochlear partition (BM with the organ of Corti) was extracted, rinsed with water, placed on the microscopic slide with a drop of glycerine and fixed with a cover slip. The reticular lamina of the organ of Corti was examined along its entire course under the light microscope with a magnification of × 400 or × 1000.

### 2.2 Ear Parameters Measurement

**Figure 2:**
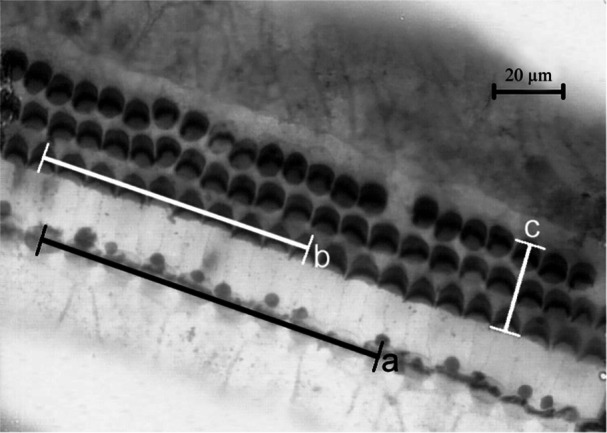
Reticular lamina of the organ of Corti of *Cryptomys pretoriae*, taken from Lange (2005). Surface specimen. Focused on stereocilia on cuticular plates of hair cells, a = measurement of 10 inner hair cells, b = measurement of 10 outer hair cells, c = measurement of the OHC triad width. Note the clarity with which individual cells can be recognized. When counting the hair cells, only sites with a regular geometric pattern were evaluated, and sites with malformations in the geometric pattern (supernumerary hair cells or cell aplasia, as seen in the third row of OHC) were excluded.

**Figure 3:**
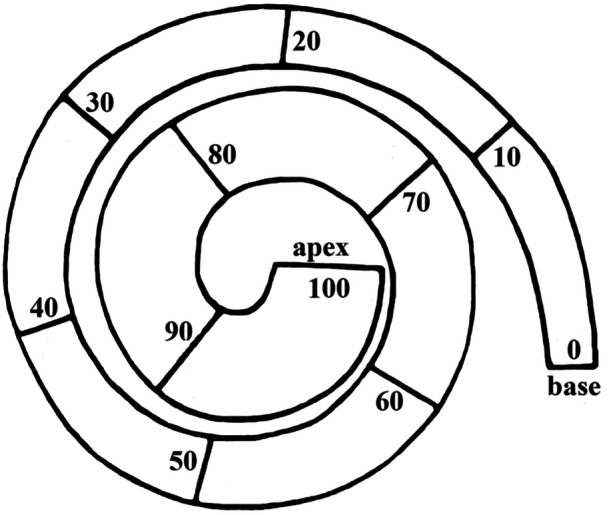
Cochlear spiral divided into ten segments of equal length from the base (0%) to the apex (100%). For clearer illustration of the principle a less coiled cochlear spiral of the Norway rat is depicted.

Each organ of Corti was examined along its entire course and each visual segment was measured using an ocular micrometer under light microscopy. The length of the BM was measured along the tunnel of Corti. The only segments of the BM not included in the further measurements were those where qualitative or quantitative evaluation of the hair‐cell population was impossible. In the present report, we focus on three parameters: the radial width of the regular three rows (i.e., the OHC in the fourth row, if present, were not considered) of cuticular plates in the outer hair cells (OHC triad) and the density of IHC and OHC. The density was measured indirectly by measuring the distance occupied by 10 hair cells in the row (Figure 2). In order to compare the cochleae with each other, each cochlear partition was then divided into 10 segments of equal length (Figure 3), and the results were expressed as average width of OHC triad and average density of hair cells per segment.

For interspecific comparison of basoapical change of the OHC triad width and densities of both IHC and OHC, the percentage difference between the base and apex were calculated for each sample as follows: ((Va‐Vb)/Vb)*100, where Va is the value at the apex and Vb is the value at the base. In cases where data for the 95% distance from the base were unavailable or unreliable, we used the values for the 85% distance from the base—that is, the higher of the two values.

### 2.3 Statistical Analyses

Statistical analyses were limited to seven species (*B. suillus*, *C. hottentotus*, *F. mechowii*, *F. whytei*, *G. capensis*, *H. argenteocinereus* and *H. glaber*), as the original dataset from Lange et al. (2005), necessary for these analyses, is no longer available. Intraspecific differences in studied traits (OHC triad width, IHC and OHC density) along the BM were tested with Generalized Least Squares marginal models (GLS) implemented in the nlme package (Pinheiro et al. 2023), followed by post‐hoc comparisons using the Tukey method implemented in the multcomp package (Hothorn et al. 2008). Each species‐specific GLS model tested the effect of the segment of the BM (categorical variable, nine levels) and included specimen identity to account for repeated measurements taken along the BM for each specimen. Post‐hoc tests were calculated for 15 comparisons, and their results were corrected using the Bonferroni correction (*α* = 0.05/15).

To examine interspecific variation in intracochlear changes of the studied parameters along the BM, we again used GLS marginal models followed by post‐hoc comparisons using Estimated Marginal Means implemented in the emmeans package (Lenth 2020). For each parameter, a model included the effects of: (1) BM segment (categorical variable), (2) bathyergid species (categorical variable, 7 levels) and (3) their interaction; specimen identity was included to account for repeated measurements taken along the BM for each specimen. The values of the width of the OHC triad and the density of IHC and OHC at each of nine points between 10 segments were compared by pair‐wise comparisons using Estimated Marginal Means. The effects of CBL on selected inner ear parameters in mole‐rat species were examined using linear regression analyses. All statistical analyses were performed in R (R Core Team 2024).

## 3 Results

### 3.1 Cochlea

**Figure 4:**
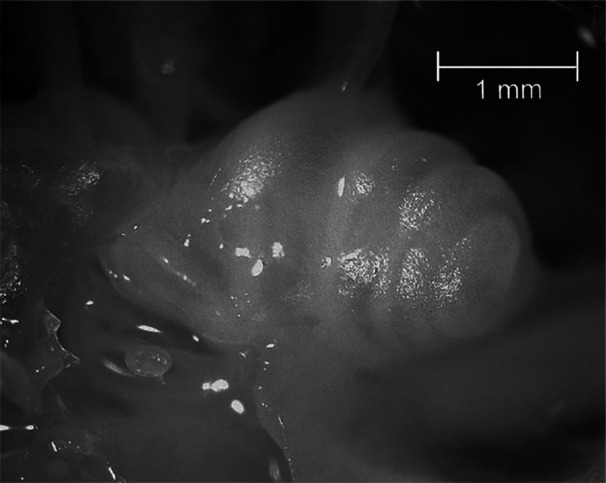
The cochlea of *Cryptomys pretoriae*. Note its tower‐like, tightly coiled shape.

The cochlea in all the species under study was thin‐walled, blunt conical, and only partly free‐standing. The lower half of the cochlea was attached to the medial wall of the middle ear cavity and was supported by several trabeculae. The number of coils exposed during the surface specimen preparation amounted to 3–3.5 coils in all the bathyergids under study, except in *B. suillus* with 4.3 cochlear coils (Table 3, Figure 4).

### 3.2 BM Length

The BM length ranged from 6.5 mm in *H. glaber* to 15.6 mm in *B. suillus* (Table 2).

**Table 2:** Number of cochlear coils, length of basilar membrane (BM) and number of outer (OHC) and inner (IHC) hair cells and their values per mm in the studied African mole‐rat species.

Species	Number of coils	BM length (mm)	IHC/mm	IHC total number	OHC/mm	OHC total number
*Bathyergus suillus*	4.3	15.6 ± 1.4	114 ± 15	1778	423 ± 32	6600
*Cryptomys hottentotus* a	3.0	9.1 ± 1.3	122 ± 12	1110	448 ± 34	4077
*Cryptomys natalensis* a	3.5	10.7 ± 0.3	120 ± 13	1284	474 ± 38	5073
*Cryptomys pretoriae* a	3.5	9.3 ± 1.5	116 ± 15	1079	445 ± 41	4140
*Fukomys anselli* a	3.5	9.3 ± 0.5	116 ± 8	1079	451 ± 23	4194
*Fukomys damarensis* a	3.5	10.4 ± 1.1	116 ± 13	1206	445 ± 51	4629
*Fukomys darlingi* a	3.3	11.9 ± 0.9	117 ± 15	1392	480 ± 36	5712
*Fukomys mechowii*	3.6	11.2 ± 0.8	114 ± 5	1277	429 ± 15	4806
*Fukomys whytei*	3.3	9.3 ± 0.4	109 ± 8	1014	425 ± 22	3954
*Georychus capensis*	3.1	11.1 ± 0.7	114 ± 5	1265	425 ± 27	4719
*Heliophobius argenteocinereus*	3.4	9.7 ± 0.2	114 ± 9	1106	399 ± 25	1290
*Heterocephalus glaber*	3.3	6.5 ± 0.1	104 ± 10	676	390 ± 24	2535

a Data published in Lange (2005).

### 3.3 IHC Density

**Figure 5:**
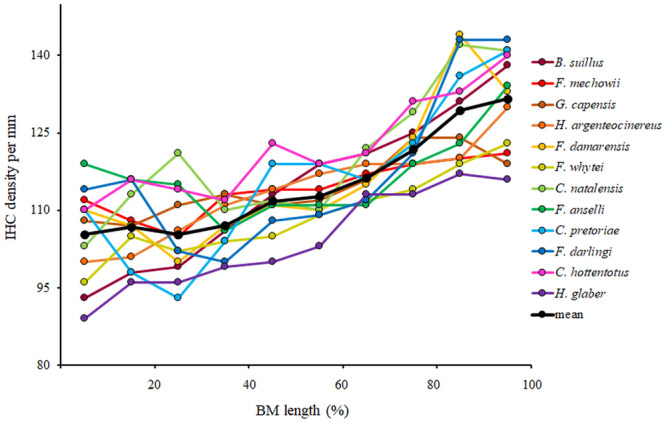
Variation in inner hair cell density (IHC) along the basilar membrane from the base (0%) to the apex (100%) in particular African mole‐rat species compared to the mean values across all the 12 studied species.

The mean IHC density in studied mole‐rats was 115 IHC/mm ranging from 104 in *H. glaber* to 122 in *C. hottentotus*. *C. hottentotus* possess significantly higher density of IHC than *H. glaber* along the whole BM length and also than *F. whytei* along most of the BM length (Supporting Information S1: 1). In examined mole‐rats, IHC density varied significantly along the BM length (Supporting Information S1: 2). It remained relatively constant (about 110 IHC/mm) over the basal 60% of the cochlear length for the majority of species and then increased to about 140 IHC/mm at the apex (Table 3, Figure 5). The total number of IHC (calculated as BM length multiplied by mean IHC density) ranged from 660 IHC in *H. glaber* to 1750 in *B. suillus*. The basoapical difference ranged between 11% in *F. mechowii* and 38% in *C. natalensis* (Table 3).

**Table 3:** Density of inner hair cells per 1 mm along the length of the organ of Corti from the base (0%) to the apex (100%).

Segment (%)	Bsui	Chot^a^	Cnat^a^	Cpre^a^	Fans^a^	Fdam^a^	Fdar^a^	Fmec	Fwhy	Gcap	Harg	Hgla	Mean	SD
5	93	110	103	110	119	110	114	112	96	108	100	89	105	9
15	98	116	113	98	116	107	116	108	105	107	101	96	107	7
25	99	114	121	93	115	100	102	105	102	111	106	96	105	8
35	106	112	110	104	106	107	100	113	104	113	111	99	107	5
45	113	123	112	119	111	111	108	114	105	111	114	100	112	6
55	119	119	110	119	111	110	109	114	109	112	117	103	113	5
65	121	121	122	116	111	115	112	117	112	116	119	113	116	4
75	125	131	129	123	119	124	121	119	114	124	119	113	122	5
85	131	133	142	136	123	144	143	120	119	124	120	117	129	10
95	138	140	141	141	134	133	143	121	123	119	130	116	132	10
Mean	114	122	120	116	116	116	117	114	109	114	114	104	115	7
SD	15	10	13	15	8	13	15	5	8	6	9	10		
BAD	34	28	38	28	13	31	25	11	25	15	28	31		

Abbreviations: BAD, basoapical difference expressed in %; Bsui, *Bathyergus suillus*; Chot, *Cryptomys hottentotus*; Cnat, *C. natalensis*; Cpre, *C. pretoriae*; CV, coefficient of variation; Fans, *Fukomys anselli*; Fdam, *F. damarensis*; Fdar, *F. darlingi*; Fmec, *F. mechowii*; Fwhy, *F. whytei*; Gcap, *Georychus capensis*; Harg, *Heliophobius argenteocinereus*; Hgla, *Heterocephalus glaber;* SD, standard deviation.

a Data published in Lange (2005).

### 3.4 OHC Density

**Figure 6:**
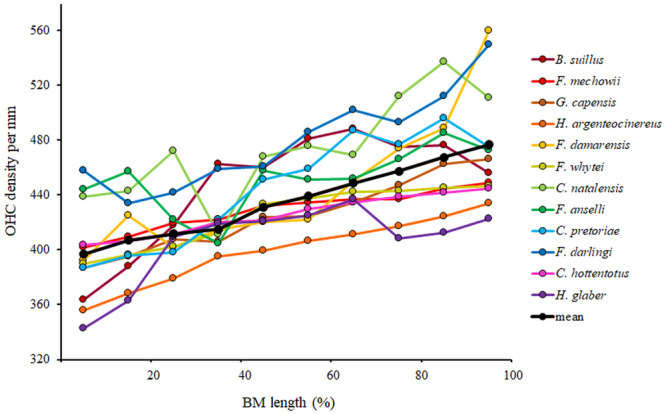
Variation in outer hair cell density (OHC) along the basilar membrane from the base (0%) to the apex (100%) in particular African mole‐rat species compared to the mean values across all the 12 studied species.

The mean OHC density in the studied mole‐rats was about 440 OHC/mm, ranging from 399 in *H. argenteocinereus* to 480 in *F. damarensis*. In examined mole‐rats, OHC density varied significantly along the length of the organ of Corti (Supporting Information S1: 2). On average, the value changed only slightly along the length of the organ of Corti, remaining relatively constant (approximately 426 OHC/mm) over the basal 40% of the cochlear length, before increasing to about 513 OHC/mm at the apex (Table 4, Figure 6). Among the tested species, the most pronounced changes along BM were found in *H. argenteocinereus* and *B. suillus* (Supporting Information S1: 2). The total number of OHC (calculated as BM length multiplied by mean OHC density) thus ranged from 2639 OHC in *H. glaber* to 6973 in *B. suillus*. The basoapical difference ranged between 9% in *F. anselli* and 28% in *C. pretoriae*.

**Table 4:** Density of outer hair cells per 1 mm along the length of the organ of Corti from the base (0%) to the apex (100%).

Segment (%)	Bsui	Chot^a^	Cnat^a^	Cpre^a^	Fans^a^	Fdam^a^	Fdar^a^	Fmec	Fwhy	Gcap	Harg	Hgla	Mean	SD
5	363	401	439	387	444	392	458	402	390	387	356	343	356	122
15	388	409	443	396	457	425	434	410	396	395	368	363	364	122
25	398	421	472	398	422	402	442	420	402	408	379	378	367	123
35	420	425	411	422	405	415	459	422	413	406	395	387	368	122
45	415	426	468	451	458	420	461	432	433	424	399	388	385	126
55	437	443	476	459	451	422	486	435	437	424	406	394	392	129
65	445	451	469	487	452	450	502	437	443	435	412	403	401	132
75	451	462	512	477	466	474	493	437	443	447	417	409	410	133
85	453	451	537	496	485	489	512	445	445	463	424	412	420	137
95	456	476	511	475	473	560	550	449	447	466	434	423	428	140
Mean	423	437	474	445	451	445	480	429	425	425	399	390	389	129
SD	32	24	38	41	23	51	36	15	22	27	25	24		
BAD	17	19	22	28	9	43	20	11	11	25	20	25		

Abbreviations: BAD, baso‐apical difference expressed in %; Bsui, *Bathyergus suillus*; Chot, *Cryptomys hottentotus*; Cnat, *C. natalensis*; Cpre, *C. pretoriae*; CV, coefficient of variation; Fans, *Fukomys anselli*; Fdam, *F. damarensis*; Fdar, *F. darlingi*; Fmec, *F. mechowii*; Fwhy, *F. whytei*; Gcap, *Georychus capensis*; Harg, *Heliophobius argenteocinereus*; Hgla, *Heterocephalus glaber;* SD, standard deviation.

a Data published in Lange (2005).

### 3.5 OHC Triad Width

**Figure 7:**
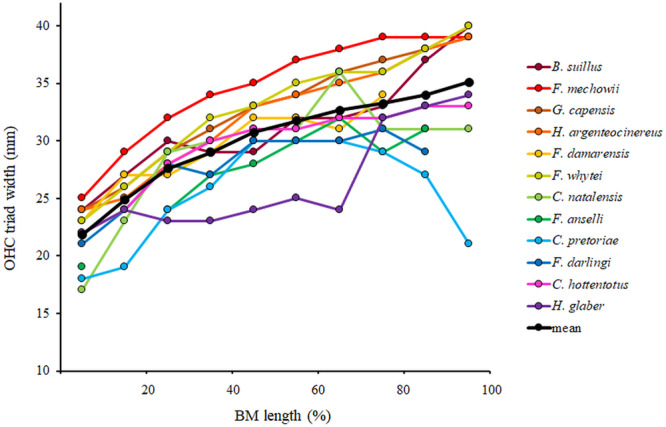
Variation in outer hair cell (OHC) triad width along the basilar membrane from the base (0%) to the apex (100%) in particular African mole‐rat species compared to the mean values across all the 12 studied species.

The OHC triad width differed significantly in all tested species (Supporting Information S1: 2). The basoapical increase in the width of the OHC triad was continuous and rather linear, though steeper along the basal half of the cochlear length than in the apical half (Figure 7, Supporting Information S1: 2). The minimum (basal) values were about 20 µm, the maximum (apical) values of the parameter reached between 30 and 40 µm in all the species, which implies the basoapical difference was more than 50% (Table 5, Figure 7).

**Table 5:** Width of the OHC triad (mean values in µm) along the length of the organ of Corti from the base (0%) to the apex (100%).

Segment (%)	Bsui	Chot^a^	Cnat^a^	Cpre^a^	Fans^a^	Fdam^a^	Fdar^a^	Fmec	Fwhy	Gcap	Harg	Hgla	Mean	SD
5	24	22	17	18	19	23	21	25	23	24	24	22	22	3
15	27	24	23	19	n.d.	27	24	29	26	26	25	24	25	3
25	30	28	29	24	24	27	28	32	29	29	28	23	28	3
35	29	30	30	26	27	29	27	34	32	31	30	23	29	3
45	29	31	31	30	28	32	30	35	33	33	33	24	31	3
55	32	31	31	30	30	32	30	37	35	34	34	25	32	3
65	32	32	36	30	32	31	30	38	36	36	35	24	33	4
75	33	32	31	29	29	34	31	39	36	37	36	32	33	3
85	37	33	31	27	31	n.a.	29	39	38	38	38	33	34	4
95	40	33	31	21	n.a.	n.a.	n.a.	39	40	39	39	34	35	6
Mean	31	30	29	25	28	29	28	35	33	33	32	26	30	3
SD	5	4	5	5	4	4	3	5	6	5	5	5		
BAD	63	52	82	50	63	n.a.	38.	54	52	54	55	72		

Abbreviations: BAD, basoapical difference expressed in %; Bsui, *Bathyergus suillus*; Chot, *Cryptomys hottentotus*; Cnat, *C. natalensis*; Cpre, *C. pretoriae*; CV, coefficient of variation; Fans, *Fukomys anselli*; Fdam, *F. damarensis*; Fdar, *F. darlingi*; Fmec, *F. mechowii*, Fwhy *F. whytei*; Gcap, *Georychus capensis*; Harg, *Heliophobius argenteocinereus*; Hgla, *Heterocephalus glaber;* n.d., no data; SD, standard deviation.

a Darta published in Lange (2005).

### 3.6 Additional OHC

**Figure 8:**
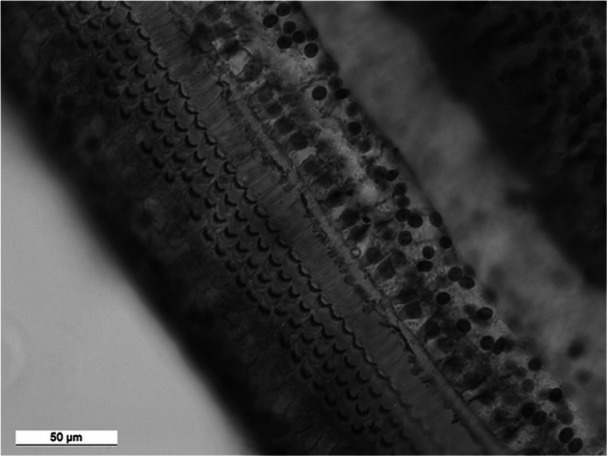
Four rows of outer hair cells in *Bathyergus suillus*.

A row of additional OHC extending across the whole visual field with the magnification of × 1000 (approx. 0.15 mm), and hence encompassing about 20 OHC, was classified as an additional OHC row (Figure 8). Additional (fourth) row of OHC was found between in about the middle (from the 4th to the 7th segment) of the BM length in three out of six studied cochleae of *B. suillus*, specifically between 35% and 65%, 25% and 45%, and 45% and 75% in each cochlea, respectively. In *H. glaber*, the fourth row of OHC was present between the 3rd and 7th segment (specifically between 25% and 55% and around 65%) in two out of the four studied cochleae.

### 3.7 Effect of Body Size on Inner Ear Parameters

**Figure 9:**
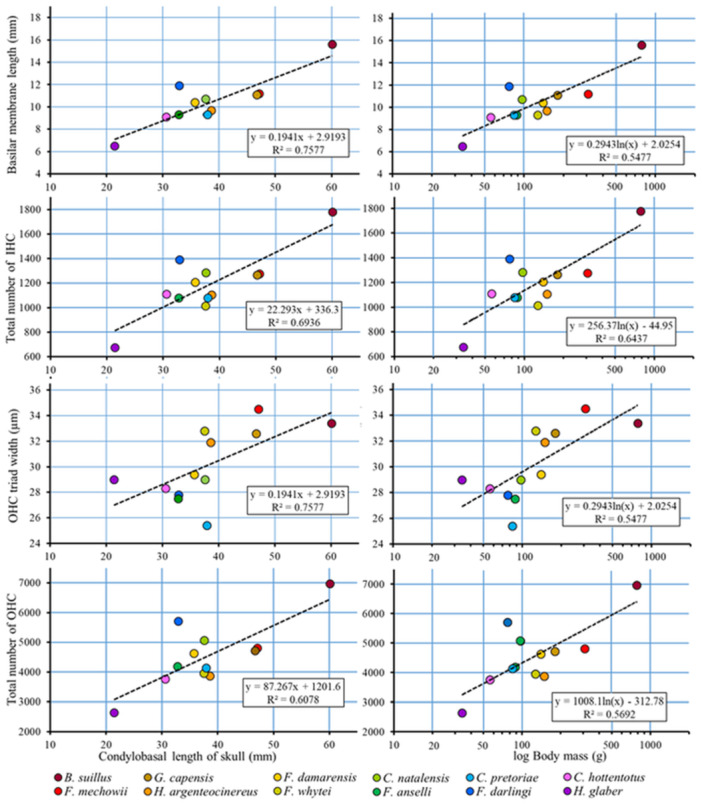
Relationship between inner ear characteristics and mole‐rat species size expressed as condylobasal length of the skull and logarithmic body mass in 12 African mole‐rat species. IHC, inner hair cells; OHC, outer hair cells.

Body size (represented here by the CBL of the skull and body mass) proved to have a significant effect on both BM length and width (represented here by the width of the OHC triad) (see Table 6 and Figure 9 for statistical details). Although body size did not influence mean IHC and OHC densities, it affected the total number of both hair cells through the BM length (Table 6, Figure 9).

**Table 6:** Results of linear regressions testing the effects of condylobasal length (CBL) and body mass (BM) on selected parameters. Statistically significant relationships after correction for multiple testing using the Benjamini‐Hochberg procedure are shown in bold, with adjusted *p* values in parentheses.

Response	Term	Estimate	Std. error	*t* value	*p* values	Rsq
**BML**	**(Intercept)**	2.92	1.37	2.14	0.06	0.733
	**CBL**	0.19	0.03	5.59	< 0.001 (0.002)	
	**(Intercept)**	23.0	2.76	8.32	< 0.001	0.356
	**CBL**	0.19	0.07	2.66	0.024 (0.060)	
Triad 5%	(Intercept)	16.7	2.87	5.82	< 0.001	0.155
	CBL	0.13	0.07	1.74	0.113 (0.189)	
Triad 85%	(Intercept)	25.20	4.69	5.37	0.0004	0.204
	CBL	0.22	0.12	1.89	0.092 (0.183)	
IHC mean	(Intercept)	112.0	5.93	18.9	< 0.001	−0.076
	CBL	0.07	0.15	0.48	0.644 (0.920)	
**IHC total**	**(Intercept)**	336.0	184.0	1.82	0.098	0.663
	**CBL**	22.3	4.69	4.76	0.001 (0.004)	
OHC mean	(Intercept)	435.0	31.0	14.0	< 0.001	−0.097
	CBL	0.12	0.79	0.16	0.880 (0.925)	
**OHC total**	**(Intercept)**	1202.0	872.0	1.38	0.198	0.568
	**CBL**	87.3	22.2	3.94	0.003 (0.009)	
Triad apex/base	(Intercept)	1.54	0.14	10.8	< 0.001	−0.102
	CBL	0.00	0.00	0.27	0.793 (0.925)	
OHC/IHC	(Intercept)	3.79	0.25	15.10	< 0.001	−0.099
	CBL	0.00	0.01	0.10	0.925 (0.925)	
**BML**	**(Intercept)**	−15.9	5.10	−3.12	0.011	0.700
	**B MASS (LOG)**	16.7	3.24	5.16	< 0.001 (0.004)	
Triad mean	(Intercept)	6.4	10.20	0.62	0.547	−0.100
	B MASS (LOG)	15.1	6.50	2.33	0.042 (0.106)	
Triad 5%	(Intercept)	6.48	10.50	0.62	0.550	0.089
	B MASS (LOG)	9.58	6.65	1.44	0.180 (0.300)	
Triad 85%	(Intercept)	5.69	16.9	0.34	0.744	0.151
	B MASS (LOG)	17.90	10.7	1.67	0.130 (0.260)	
IHC mean	(Intercept)	96.60	20.3	4.76	0.001	−0.019
	B MASS (LOG)	11.50	12.9	0.89	0.393 (0.561)	
**IHC total**	**(Intercept)**	−1889.00	648.0	−2.91	0.016	0.663
	**B MASS (LOG)**	1959.00	412.0	4.76	0.001 (0.004)	
OHC mean	(Intercept)	401.00	108.0	3.69	0.004	−0.086
	B MASS (LOG)	24.70	68.90	0.36	0.727 (0.808)	
**OHC total**	**(Intercept)**	−7392.00	3113.00	−2.37	0.039	0.556
	**B MASS (LOG)**	7595.00	1977.00	3.84	0.003 (0.011)	
Triad apex/base	(Intercept)	1.38	0.50	2.77	0.022	−0.090
	B MASS (LOG)	0.13	0.32	0.42	0.687 (0.808)	
OHC/IHC	(Intercept)	3.83	0.88	4.34	0.001	−0.100
	B MASS (LOG)	−0.01	0.56	−0.02	0.984 (0.984)	

Abbreviations: B MASS (LOG), logarithm of body mass; BML, basilar membrane length; CBL, condylobasal length; IHC, inner hair cell density; OHC, outer hair cell density; Rsg, partial R‐squared (proportion of explained variation); Triad mean, mean width of the OHC triad; Triad 5%, 85%, OHC triad width measured at 5% and 85% of BML from the base; Triad apex/base, ratio of TR 85% to TR 5%; estimate, estimated values of the intercept and slope; *t*‐value, t‐statistic testing the significance of the given parameter.

## 4 Discussion

The analysis of inner ear morphology across African mole‐rats from all six genera revealed shared traits typical for subterranean species: a tower‐shaped cochlea with a relatively high number of coils, slow widening of the OHC triad width from the base towards the apex, and the highest density of hair cells in the apical part of the organ of Corti. An additional (i.e., fourth) row of OHC in the central part of the BM in *B. suillus* and *H. glaber* was present in approximately 50% of individuals, suggesting that it is neither an abnormal nor a species‐specific trait.

### 4.1 Cochlea

The number of cochlear turns was about 3.5 in all studied bathyergid species, with the notable exception of *B. suillus*, which exhibited about 4.3 turns. While Mason et al. (2016) reported 2.7 cochlear turns in *H. glaber*, we observed 3.3 turns in this species. The difference may be due to different methods of estimation rather than to real intraspecific variability in cochlear coiling in the *H. glaber*. Generally, the number of cochlear turns is species‐specific and is roughly even genus‐ and family‐specific. The same number of turns may be found in different species from quite unrelated families and orders. Multiple lines of phylogenetic, physiological and morphological comparative evidence across a wide array of mammalian taxa suggest that, at least within lower taxa (i.e., below an order), an increased number of cochlear coils represents an apomorph (i.e. derived, secondary, advanced) trait (see Burda 1979, 1988; Fleischer 1973).

Fleischer (1978) pointed out a correlation between a higher number of coils (conical, tower‐like cochlea) and low‐frequency hearing on the one hand and a lower number of coils (flat cochlea) and high‐frequency hearing on the other hand in the cetaceans. Our studies of cochleae in subterranean and fossorial mammals (Burda et al. 1988; Lange et al. 2004; Pleštilová et al. 2021) confirm Fleischer's observations, provided the comparison is made between related species which diverged into different acoustic environments. Conical, highly coiled cochleae are, however, typical also for hystricomorph rodents (Fleischer 1973; Mason et al. 2016; Pye 1977), the suborder to which bathyergids belong. While coiling *per se* may enhance frequency discrimination (separation of frequencies) along the cochlear duct (Růžek and Voldřich 1977), the number of cochlear coils does not seem to have a direct relation to the auditory range (regarding the number of octaves) and hearing capabilities. The effect of curvature is particularly prominent in low‐frequency processing regions (Manoussaki et al. 2006).

### 4.2 BM

Although the number of cochlear coils (apart from *B. suillus*) varied only slightly (3.3 ± 0.2), there were significant differences in the respective lengths of the BM among the species examined, and the BM length correlated with CBL of the skull (CBL) and the body mass (Table 6, Figure 9). While CBL may be used as an explanatory factor for (within) intrafamilial comparison, it is not so suitable—because of the different skull morphotypes—for correlative comparisons between representatives of higher (than family) taxa. It may be of interest that although *F. darlingi* in our study sample originated from the Goromonzi population characterized by a small body size (Šumbera et al. 2023), its BM length (but, interestingly, also OHC triad width—cf. Figure 7) corresponded to a much larger body size characterizing other populations of this species, indicating thus that a smaller body size in that population might be an apomorph (i.e., derived secondary, advanced) feature.

**Figure 10:**
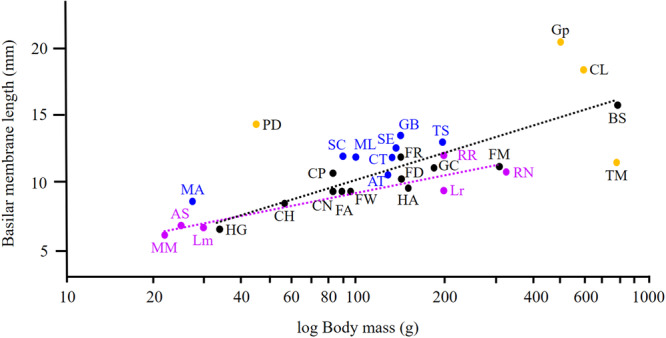
Relationship between basilar membrane length and animal body size. Violett: generalized epigeic Muridae MM, *Mus musculus*, AS *Apodemus sylvaticus*, Lm Laboratory mouse (NMRI), Lr Laboratory rat (Wistar), RR *Rattus rattus*, RN *Rattus norvegicus* (wild); Blue: fossorial and subterranean rodents (without bathyergids) MA *Microtus arvalis*, SC *Spalacopus cyanus*, ML *Meriones lybicus*, AT *Arvicola terrestris*, CT *Ctenomys talarum*, SE *Spalax ehrenbergi*, GB *Geomys bursarius*, TS *Tachyoryctes splendens*, TM *Tachyoryctes macrocephalus*; Black: Bathyergidae HG *Heterocephalus glaber*, CH *Cryptomys hottentotus*, CP *C. pretoriae* CN *C. natalensis*, FA *Fukomys anselli*, FW *F. whytei*, FD *F. damarensis*, FR *F. darlingi*, HA *Heliophobius argenteocinereus*, GC *Georychus capensis*, FM *F. mechowii*, BS *Bathyergus suillus*; ochre: (other rodent species from which BML is known, yet which apparently do not fit into the proposed body weight/BML relationship) PD *Pachyuromys duprasi*, Gp *Guinea pig*, CL *Chinchilla laniger*. Data based: on this study, Begall and Burda (2006), Burda (1984), Burda (own unpubl. data), Burda et al. (1989, 1992), Lange et al. (2004), Pleštilová et al. (2021), Schleich et al. (2006).

Comparing with known data on BM length (obtained by the same preparation method) in unrelated rodent species reveals that BM length correlates primarily with body size (represented here by body mass and CBL), whereby the y‐axis intercept and the slope of the regression line are taxonomically determined (Figures 9 and 10). Note that interfamilial comparisons relating BM length (or any other trait) to body size have to be based rather on body mass than CBL because CBL is influenced by the skull shape, and this, in turn, is subservient to ecology, and hence strongly varying among families.

There are indications, noted already by previous authors (Begall and Burda 2006; Burda et al. 1989, 1992; Lange et al. 2004; Pleštilová et al. 2021; Schleich et al. 2006) that subterranean rodents tend to have longer BM than their epigeic relatives. This relationship becomes less obvious if we extend the sample size for some further rodent species, notably the guinea pig *Cavia porcellus*, chinchilla *Chinchilla lanigera*, fat‐tailed gerbil *Pachyuromys duprasi* and giant root rat *Tachyoryctes macrocephalus* (Figure 10).

Drawing on experimental cochlear mechanics (reviewed i.e., in Olson et al. 2012), we propose that elongation of the BM may have two different functional implications: (1) an overall extension of the frequency range or (2) an expansion of (biologically relevant) frequency bands/octaves and thus improvement of differential frequency and/or intensity discrimination. In both scenarios, it is the greater number of hair cells that can be accommodated by longer BM that is responsible for improved hearing rather than the mere length of the BM.

### 4.3 IHC Density

The density of IHC increased towards the apex in all studied African mole‐rat species (Figure 5). This is a typical trend in subterranean rodents (Lange 2005; Pleštilová et al. 2016, 2019), whereas epigeic rodents tend to have maxima of IHC in the basal or central part of the cochlea (Burda et al. 1988; Ding et al. 2001). In fossorial species, the pattern of IHC density is not uniform. In the coruro (*S. cyanus*) and Talas tuco‐tuco (*Ctenomys talarum*), IHC density reaches a maximum in the apical part of the cochlea (Begall and Burda 2006; Schleich et al. 2006). The common vole (*Microtus arvalis*) and the water vole (*Arvicola terrestris*) have one maximum in the basal and another in the apical part with a minimum in the middle part of the cochlea (Lange et al. 2004). In root rats *Tachyoryctes splendens* and *T. macrocephalus*, the IHC density maxima are in the central part of the cochlea (Pleštilová et al. 2021).

The values of IHC density are very similar for all bathyergids as well, with an exception in *C. hottentotus*, which has significantly denser IHC than *H. glaber* and *F. whytei* (Supporting Information S1: 1). This can make a small difference in hearing sensitivity, which is very similar in all bathyergids studied so far (Brückmann and Burda 1997; Caspar et al. 2021; Gerhardt et al. 2017; Heffner and Heffner 1990, 1992, 1993; Kössl et al. 1996; Müller and Burda 1989; Müller et al. 1992; Pyott et al. 2020).

Since hearing of subterranean rodents is tuned to low frequencies and low frequencies are processed in the apical cochlear regions, our findings support the previously found correlations between the IHC density distribution and tuning of the cochlea in epigeic rodents (Burda 1984; Burda and Voldřich 1980).

### 4.4 OHC Density

The density of OHC increases towards the apex in most African mole‐rat species, with the exception of *H. glaber* and *B. suillus*, which both have the maximum in the central part of the organ of Corti (Figure 6). In this central part, *B. suillus* possessed denser OHC than *H. argenteocinereus* and, surprisingly, to a lesser extent also than *H. glaber* (Supporting Information S1: 1). Similar density of the OHC in the central part was found also in *F. darlingi*, *C. pretoriae* and *C. natalensis*, which were not involved in the statistical comparison (Figure 6). The maximum OHC density situated approximately in the middle of the organ of Corti is a common mammalian pattern (Burda et al. 1988; Ding et al. 2001), whereas having a maximum OHC density in the apical part is typical for subterranean and fossorial rodents (Burda et al. 1989; Lange 2005; Pleštilová et al. 2016, 2021).

Since the cochlea is tonotopically organized (reviewed in Olson et al. 2012), the highest OHC density in its central part can be related to a higher sensitivity or better frequency resolution for higher frequencies (Burda 1984; Burda and Voldřich 1980). This is consistent with the known hearing abilities of African mole‐rats, as *H. glaber* exhibits peak sensitivity around 4 kHz, whereas other studied species show maximal sensitivity near 1 kHz (Brückmann and Burda 1997; Gerhardt et al. 2017; Heffner and Heffner 1993; Okanoya et al. 2018). Such differences in acoustic perception are particularly important for social communication and coordination within *H. glaber* groups (Barker et al. 2021). Unfortunately, there is no information concerning the hearing abilities in the *B. suillus*, so we can only speculate that the best sensitivity of hearing may occur at higher frequencies than in other African mole‐rats.

Hearing abilities of subterranean mammals are closely related to the species' vocal communication, which is influenced mainly by the acoustic properties of burrows and the body mass of the animal (Bradbury and Vehrencamp 1998; Credner et al. 1997; Hrouzková and Schleich 2023). Consequently, an ability to hear relatively high frequencies in *B. suillus* might be caused by its occasional aboveground activity, whereas in *H. glaber* it can be caused by vocalization constraints given by its small body size, as the frequency of vocalization is negatively correlated with the body mass of the animals (Fletcher 2004).

### 4.5 Width of OHC Triad

**Figure 11:**
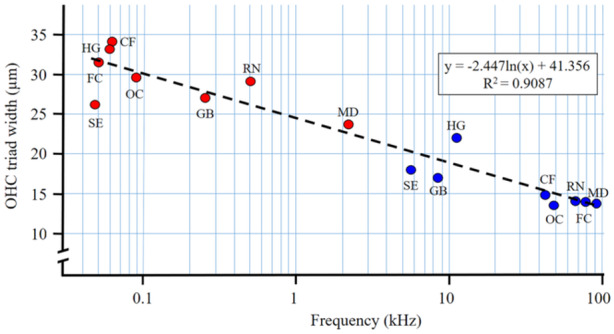
OHC triad width at the apex (red dots) and base (blue dots) plotted against low and high‐frequency hearing cutoffs in mammalian species for which these values are known. HG *Heterocephalus glaber*, FC domestic cat, CF domestic dog, OC domestic rabbit, GB *Geomys bursarius*, RN laboratory rat, SE *Spalax ehrenbergi*, MD laboratory mouse. Data on OHC triad width originate from our labs, data on hearing range from the lab of H. E. and R. S. Heffner (cf. Table 7).

It has been suggested that the ratio between apical and basal values contains information on frequency distribution along the BM (see Burda 1985; Burda et al. 1988; Burda and Branis 1988). To test this assumption, we have plotted the basal and apical values of the OHC triad width against known high‐frequency and low‐frequency hearing cutoffs in animal species for which (1) OHC triad width was established by the same method of study of surface specimens of cochlear partitions in our labs, and (2) at the same time also hearing thresholds have been established by a comparable method in the same lab (H. E. and R. S. Heffner's lab) (see Table 7, Figure 11).

**Table 7:** Hearing range and OHC triad width values in species for which also behavioural audiograms are available. Low‐frequency hearing cutoff is here associated with apical values of OHC triad width, while high‐frequency hearing cutoff is associated with basal values of the OHC triad width.

Frequency audible at 60 dB SPL (Hz)		OHC triad width (µm)	Species	Ref. hearing	Ref. OHC triad
Lowest	65	33	*Heterocephalus glaber*	Heffner and Heffner (1993)	This paper
Highest	11,500	22			
Lowest	530	29	Laboratory rat	Heffner et al. (1994)	Burda et al. (1988)
Highest	68,000	14			
Lowest	2300	24	Laboratory mouse	Heffner and Masterton (1980)	Burda et al. (1988)
Highest	92,000	14			
Lowest	52	26	*Nanospalax ehrenbergi*	Heffner and Heffner (1992)	Burda et al. al. 1989
Highest	5900	18			
Lowest	270	27	*Geomys bursarius*	Heffner and Heffner (1990)	Burda unpubl.
Highest	8700	17			
Lowest	96	30	Domestic rabbit	Heffner and Masterton (1980)	Burda and Branišunpubl.
Highest	49,000	14			
Lowest	55	31	Domestic cat	Heffner and Heffner (1985)	Burda unpubl.
Highest	79,000	14			
Lowest	67	34	Domestic dog	Heffner (1983)	Burda and Branišunpubl.
Highest	44,000	15			

**Figure 12:**
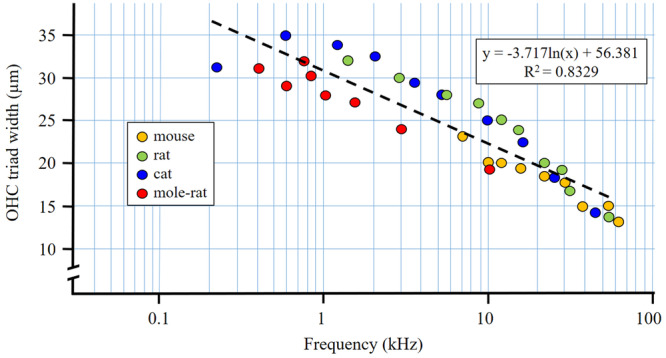
Correlation between the OHC triad width and frequencies registered at particular positions along the basilar membrane according to the published tonotopy maps. See Table 8 for detail.

Another approach to correlate OHC triad width with a frequency registered at a given position along the cochlear spiral has been based on measurements done in species for which place‐frequency (i.e., tonotopy) maps are available (Table 8, Figure 12).

**Table 8:** Hearing range and OHC triad width values (in µm) in species for which also frequency (in Hz)‐place (i.e., tonotopy) maps are available.

Species References: OHC triad tonotopy map	Parameters	Position on the basilar membrane from base to apex (%)									
		5	15	25	35	45	55	65	75	85	95	
Laboratory mouse Burda et al. 1988 Müller et al. 2005	OHC triad	13	15	15	17	18	19	20	20	23	20	
	Frequency	X	54,000	39,000	30,000	22,000	16,000	12,000	10,000	7000	x	
Laboratory rat Burda et al. 1988 Müller 1991	OHC triad	14	16	19	20	23	25	27	28	30	32	
	Frequency	55,000	37,000	29,000	22,000	16,000	12,000	8600	5900	2900	1400	
Domestic cat Burda unpubl. Liberman 1982	OHC triad	14	17	22	25	28	29	32	34	35	31	
	Frequency	45,000	27,000	17,000	10,000	5800	3500	2000	1200	580	220	
*Fukomys anselli* this paper Müller et al. (1992)	OHC triad	19	x	24	27	28	30	32	29	31	x	
	Frequency	12,000	6500	3000	1500	1000	850	750	580	400	120	

**Figure 13:**
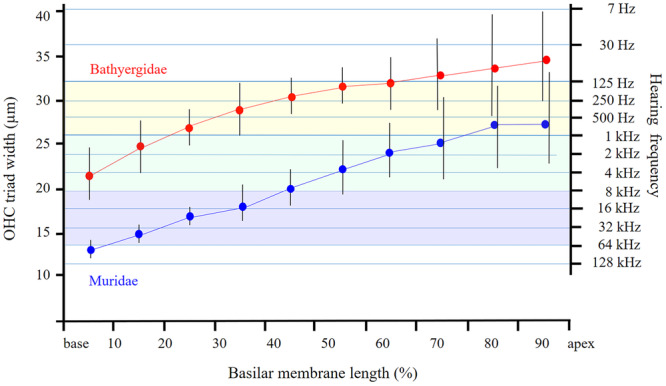
Projection of the OHC triad width changes along the cochlear partition upon the estimated tonotopy in mole‐rats (Bathyergidae, this paper, in red) and mice and rats (Muridae, Burda et al. 1988, in blue). Mean values and standard deviations are plotted. Each of the three coloured overlapping bands ranges over three octaves. Note that the octaves 1−4 kHz (green band) are represented in the apical half of the basilar membrane in murids but in the basal quarter in the bathyergids, while the low‐frequency octaves (yellow band) spread over almost half of the basilar membrane in bathyergids and are underrepresented in murids. Higher frequencies (blue band) are out of the hearing range of mole‐rats.

The analysis has confirmed a highly significant correlation between the OHC triad width and tonotopy, i.e. frequency represented at the given place at the Corti organ. Comparison of mean values of the OHC triad width along the Corti organ in the bathyergid species under present study with values in mice and rats (Burda et al. 1988) and projection of expected tonotopical values (Figure 13) reveals that overlap of the same values corresponds to overlap in perceived sound frequencies (Kössl et al. 1996; Müller 1991; Müller et al. 1992).

### 4.6 Additional OHC

Supernumerary ectopic OHC, occurring individually or even forming short segments of the fourth or even fifth OHC row, were described in apical parts of the Corti organ in humans (Rask‐Andersen et al. 2017; Retzius 1884) and diverse mammalian species such as soricids (Burda 1978), guinea‐pigs (Úlehlová 1975), monkeys (Braniš and Burda 1994), domestic pigs (Glueckert et al. 2005; Lovell and Harper 2007) and in subterranean and fossorial rodents, such as *N. ehrenbergii* and *Arvicola terrestris* (Bruns et al. 1988; Lange et al. 2004). A relatively long segment of an additional row of OHC in the central part of BM was observed only in the Gansu zokor (*E. cansus*) (Pleštilová et al. 2019). It should be pointed out that the common denominator for the occurrence of additional OHC was the cochlear regions registering lower hearing frequencies. The geometric pattern of the mammalian Corti organ, which is otherwise remarkably regular, is often disrupted in the apical regions of the cochlea. In general, it appears that the wider the organ of Corti is, the more it allows the presence of ectopic hair cells. As the OHCs actively enhance hearing of the low acoustic pressure level sounds, their number may directly influence the hearing threshold (Chen et al. 2008; Ryan and Dallos 1975; Wang et al. 2016). However, studies based on the selective dysfunction of hair cells show that the number of cells in the additional row has to be relatively high to affect hearing (Chen et al. 2008; Ryan and Dallos 1975). This raises the question, whether the segment of a fourth OHC row observed in two bathyergid species is long enough (especially in *H. glaber*) to have a functional effect. Since the presence of the additional row of OHC was observed in several individuals from both species, its adaptive significance is unclear. Principally, segments of an additional row of OHC may enhance sensitivity in low‐frequency detection, yet they may also simply represent a developmental defect (cf. review by Eliott et al. 2018) in a low‐frequency processing cochlear region where a strong geometrical pattern is probably of less functional importance and thus selection pressure for its preservation is weakened.

### 4.7 Effect of Body Size

Finding that BM length and the width of the OHC triad in the bathyergid mole‐rats are correlated with body size is consistent with the findings in mice and rats (Burda et al. 1988). This may represent a general principle that among mammals which are phylogenetically closely related, exhibit the same (or very similar) morphological habitus and occupy the same (or very similar) acoustic environment, the differences in the morphology of the cochlea of the inner ear are mainly quantitative and reflect the differences in body size. The same rule applies also to the middle ear (Burda et al. 1992). Since the BM length is decisive for the total number of hair cells, it also affects positively hearing sensitivity and frequency resolution capacities.

## 5 Conclusions and Future Perspectives

Our comparative analysis of cochlear morphology across all six genera of African mole‐rats highlights both shared adaptations to a subterranean lifestyle and subtle interspecific differences likely shaped by evolutionary history and ecological factors. Common traits, such as a conical, moderately coiled cochlea, apically increasing IHC density, and a generally slow basoapical widening of the OHC triad, reflect adaptations for enhanced sensitivity to low‐frequency sounds typical of burrow acoustics. The presence of additional OHC rows in *B. suillus* and *H. glaber*, observed in multiple individuals, may further contribute to the sensitivity, although their functional significance remains uncertain.

Although BM width can be inferred from the width of the OHC triad, critical information on BM thickness and the collagen fibre layer, key determinants of BM stiffness and cochlear travelling wave mechanics, is lacking. Consequently, we cannot precisely map the tonotopic position of each frequency along the cochlear duct or define the exact hearing range of these species. Nevertheless, our results demonstrate that OHC triad width provides a valuable proxy for estimating fundamental hearing capabilities. Future integration of anatomical data with behavioural audiograms and physiological measurements would allow more precise predictions of frequency sensitivity and further clarify the evolutionary and ecological drivers of auditory specialization in African mole‐rats.

## Author Contributions

**Lucie Svačinová:** writing – review and editing, writing – original draft, revision, conceptualization, data curation, investigation, visualization. **Simone Lange:** writing – review and editing, data curation, investigation. **Matěj Lövy:** writing – review and editing, software, methodology, formal analysis, data curation. **Barbora Konopová:** writing – review and editing, data curation, investigation. **Nigel Charles Bennett:** writing – review and editing, resources, funding acquisition, investigation. **Daniel William Hart:** writing – review and editing, resources, investigation. **Radim Šumbera:** writing – review and editing, conceptualization, resources, funding acquisition, validation, supervision. **Hynek Burda:** writing – review and editing, writing – original draft, revision, conceptualization, methodology, investigation, data curation, formal analysis, visualization, validation, supervision.

## Ethics Statement

All procedures involving animals were conducted in accordance with institutional and national guidelines for the ethical treatment of wildlife. The Animal Use and Care Committee of the University of Pretoria evaluated and approved all experimental protocols under the following ethics clearance numbers: NAS071‐2020, NAS209‐2021, and NAS072‐2023. Additional authorisation was granted under the Department of Agriculture, Forestry and Fisheries (DAFF) Section 20 approvals 12/11/1/1/8 (2002 LH) and SDAH‐Epi‐23041710072. Fieldwork and the capture of animals were conducted with the permission of all landowners and under permits issued by the respective provincial conservation authorities: KwaZulu‐Natal (KZN‐1595/2020), Western Cape (WC‐CN44‐87‐18093), and Gauteng (CPB1‐0524).

## Conflicts of Interest

The authors declare no conflicts of interest.

## Supporting information

Supplement‐S1S2.

## Data Availability

The authors have nothing to report.
