# Supplementary material for: The Ear in Subterranean Rodents Revisited: Cochlear Hair‐Cell Populations in African Mole‐Rats (Bathyergidae)
Source: J Morphol. 2025 Dec 17;286(12):e70106. doi: 10.1002/jmor.70106 (PMC12710458; doi:10.1002/jmor.70106)
Supplement: Supplementary file 1 — Supplement‐S1S2. [file JMOR-286-e70106-s001.docx]

**Supplement S1.** Comparison of the changes of densities of inner hair cells (IHC), outer hair cells (OHC), and the width of the OHC triad along the 10 % segments of the basilar membrane among African mole-rat species. The F- and p-values are provided; the numbers in parentheses indicate the degrees of freedom (DF).

| **Inner ear parameter** | **Predictor** | **F-value** | **P-value** |
| --- | --- | --- | --- |
| Inner hair cells density | Segments of basilar membrane ^(8)^ | 65.45 | <0.0001 |
|  | Species ^(6)^ | 10.57 | <0.0001 |
|  | Interaction Segments × Species | 1.43 | 0.0439 |
| Outer hair cells density | Segments of basilar membrane ^(8)^ | 26.00 | <0.0001 |
|  | Species ^(6)^ | 4.56 | 0.0002 |
|  | Interaction Segments × Species | 1.80 | 0.0024 |
| Width of the OHC triad | Segments of basilar membrane ^(8)^ | 77.02 | <0.0001 |
|  | Species ^(6)^ | 2.61 | 0.0183 |
|  | Interaction Segments × Species | 1.53 | 0.0216 |

**Supplement S1.** Results of pairwise comparisons of inner ear parameters (density of inner hair cells, outer hair cells and the width of three rows of outer hair cells, respectively) among seven species of African mole-rats. Comparisons were performed within each 10 % segment along the basilar membrane length. P-values were adjusted for multiple testing using Tukey’s HSD method, as implemented in the emmeans package in R.

**Inner hair cells density**

| **Species** | **10 %** | **20 %** | **30 %** | **40 %** | **50 %** | **60 %** | **70 %** | **80 %** | **90 %** |
| --- | --- | --- | --- | --- | --- | --- | --- | --- | --- |
| *B. suillus - C. hottentotus* | **0.0014** | **0.0486** | 0.2088 | 0.1410 | 0.6169 | 0.2597 | 0.1300 | **0.0210** | 0.4020 |
| *B. suillus - F. mechowii* | **0.0272** | 0.9674 | 0.9567 | 0.8741 | 0.9990 | 0.9237 | 0.9980 | 0.9941 | 0.8411 |
| *B. suillus - F. whytei* | 0.9157 | 0.9999 | 0.9985 | 0.9461 | 0.1124 | 0.7786 | 0.9858 | 0.2268 | **0.0154** |
| *B. suillus - G. capensis* | 0.4158 | 0.5549 | 0.8695 | 1.0000 | 1.0000 | 0.9990 | 0.9997 | 0.9992 | 0.4591 |
| *B. suillus - H. argenteocinereus* | 0.9974 | 1.0000 | 0.9898 | 1.0000 | 0.9977 | 0.8962 | 1.0000 | 1.0000 | 0.9985 |
| *B. suillus - H. glaber* | 0.4499 | 0.5968 | 0.4801 | **0.0321** | 0.0679 | 0.6640 | 0.5191 | 0.7073 | **0.0040** |
| *C. hottentotus - F. mechowii* | 0.9993 | 0.5729 | 0.9066 | 0.9373 | 0.4192 | 0.9661 | 0.5537 | **0.0077** | **0.0345** |
| *C. hottentotus - F. whytei* | **0.0001** | 0.2097 | 0.1173 | **0.0189** | **0.0010** | **0.0121** | **0.0351** | **˂0.0001** | **˂0.0001** |
| *C. hottentotus - G. capensis* | 0.6372 | 0.9718 | 0.9737 | 0.3839 | 0.8040 | 0.7051 | 0.0998 | **0.0146** | **0.0050** |
| *C. hottentotus - H. argenteocinereus* | **0.0380** | 0.0844 | 0.6820 | 0.1892 | 0.3700 | 0.9784 | 0.2956 | 0.0651 | 0.2348 |
| *C. hottentotus - H. glaber* | **˂0.0001** | **0.0004** | **0.0018** | **˂0.0001** | **0.0005** | **0.0066** | **0.0011** | **0.0003** | **˂0.0001** |
| *F. mechowii - F. whytei* | **0.0023** | 0.9974 | 0.8161 | 0.3727 | 0.4146 | 0.2397 | 0.8857 | 0.7185 | 0.4806 |
| *F. mechowii - G. capensis* | 0.9253 | 0.9854 | 1.0000 | 0.9705 | 0.9979 | 0.9974 | 0.9783 | 1.0000 | 0.9976 |
| *F. mechowii - H. argenteocinereus* | 0.2032 | 0.9646 | 0.7002 | 0.8673 | 1.0000 | 1.0000 | 0.9997 | 0.9945 | 0.9906 |
| *F. mechowii - H. glaber* | **0.0001** | 0.1972 | 0.1245 | **0.0020** | 0.3059 | 0.1690 | 0.3112 | 0.9829 | 0.2607 |
| *F. whytei - G. capensis* | 0.0710 | 0.8186 | 0.6690 | 0.9042 | 0.1470 | 0.5816 | 0.9998 | 0.6013 | 0.8310 |
| *F. whytei - H. argenteocinereus* | 0.7063 | 0.9998 | 1.0000 | 0.9829 | 0.4614 | 0.2073 | 0.9807 | 0.3033 | 0.1259 |
| *F. whytei - H. glaber* | 0.9892 | 0.5162 | 0.8652 | 0.4551 | 1.0000 | 1.0000 | 0.9598 | 0.9906 | 0.9998 |
| *G. capensis - H. argenteocinereus* | 0.8475 | 0.5947 | 0.5357 | 0.9998 | 0.9957 | 0.9949 | 0.9991 | 0.9991 | 0.8543 |
| *G. capensis - H. glaber* | **0.0079** | **0.0273** | 0.0675 | **0.0366** | 0.0953 | 0.4678 | 0.8358 | 0.9536 | 0.6045 |
| *H. argenteocinereus - H. glaber* | 0.2489 | 0.7539 | 0.9366 | 0.0930 | 0.3469 | 0.1439 | 0.5488 | 0.7596 | **0.0487** |

**Outer hair cells density**

| **Species** | **10 %** | **20 %** | **30 %** | **40 %** | **50 %** | **60 %** | **70 %** | **80 %** | **90 %** |
| --- | --- | --- | --- | --- | --- | --- | --- | --- | --- |
| *B. suillus - C. hottentotus* | 0.3498 | 0.9842 | 0.5597 | **0.0231** | 0.0519 | 0.7309 | 0.4359 | 0.9774 | 1.0000 |
| *B. suillus - F. mechowii* | 0.7822 | 0.8484 | 0.8937 | 0.1034 | 0.2738 | 0.8375 | 0.6986 | 0.9962 | 1.0000 |
| *B. suillus - F. whytei* | 0.9569 | 1.0000 | 0.3490 | 0.1320 | 0.4069 | 0.8837 | 0.8781 | 0.9983 | 0.9999 |
| *B. suillus - G. capensis* | 0.9987 | 0.9983 | 0.4071 | 0.0842 | 0.1332 | 0.9412 | 0.8186 | 0.9998 | 0.9252 |
| *B. suillus - H. argenteocinereus* | 0.9133 | 0.5036 | **0.0063** | **0.0001** | **0.0022** | **0.0463** | **0.0197** | 0.7444 | 0.9665 |
| *B. suillus - H. glaber* | 0.3140 | 0.3114 | 0.6974 | 0.1849 | **0.0003** | **0.0048** | **0.0071** | 0.7612 | 0.8735 |
| *C. hottentotus - F. mechowii* | 0.9993 | 0.9971 | 0.9997 | 1.0000 | 0.9996 | 1.0000 | 1.0000 | 1.0000 | 0.9999 |
| *C. hottentotus - F. whytei* | 0.9702 | 0.9993 | 0.9985 | 0.9984 | 0.9949 | 1.0000 | 0.9984 | 1.0000 | 1.0000 |
| *C. hottentotus - G. capensis* | 0.8100 | 1.0000 | 0.9995 | 1.0000 | 1.0000 | 0.9999 | 0.9997 | 0.9141 | 0.8796 |
| *C. hottentotus - H. argenteocinereus* | **0.0453** | 0.1387 | 0.3682 | 0.4646 | 0.8412 | 0.6391 | 0.7094 | 0.9905 | 0.9841 |
| *C. hottentotus - H. glaber* | **0.0016** | 0.0646 | 1.0000 | 0.9992 | 0.5152 | 0.2066 | 0.4973 | 0.9923 | 0.9206 |
| *F. mechowii - F. whytei* | 0.9996 | 0.9629 | 0.9801 | 0.9937 | 1.0000 | 1.0000 | 0.9999 | 1.0000 | 0.9992 |
| *F. mechowii - G. capensis* | 0.9798 | 0.9920 | 0.9889 | 1.0000 | 0.9999 | 1.0000 | 1.0000 | 0.9721 | 0.9819 |
| *F. mechowii - H. argenteocinereus* | 0.2285 | 0.0654 | 0.2680 | 0.4491 | 0.6936 | 0.7123 | 0.6845 | 0.9838 | 0.9446 |
| *F. mechowii - H. glaber* | **0.0212** | **0.0297** | 0.9999 | 1.0000 | 0.3777 | 0.2905 | 0.4898 | 0.9863 | 0.8392 |
| *F. whytei - G. capensis* | 0.9995 | 1.0000 | 1.0000 | 0.9968 | 0.9983 | 1.0000 | 1.0000 | 0.9819 | 0.8533 |
| *F. whytei - H. argenteocinereus* | 0.4583 | 0.4541 | 0.7852 | 0.8598 | 0.5521 | 0.6517 | 0.4812 | 0.9746 | 0.9976 |
| *F. whytei - H. glaber* | 0.0674 | 0.2858 | 0.9989 | 0.9702 | 0.2603 | 0.2434 | 0.3036 | 0.9782 | 0.9783 |
| *G. capensis - H. argenteocinereus* | 0.7386 | 0.3071 | 0.7339 | 0.4992 | 0.8672 | 0.5460 | 0.5591 | 0.6130 | 0.5104 |
| *G. capensis - H. glaber* | 0.1850 | 0.1761 | 0.9996 | 0.9999 | 0.5844 | 0.1765 | 0.3699 | 0.6303 | 0.3315 |
| *H. argenteocinereus - H. glaber* | 0.9638 | 1.0000 | 0.4693 | 0.3085 | 0.9990 | 0.9939 | 0.9999 | 1.0000 | 1.0000 |

**Three rows of outer hair cells**

| **Species** | **10 %** | **20 %** | **30 %** | **40 %** | **50 %** | **60 %** | **70 %** | **80 %** | **90 %** |
| --- | --- | --- | --- | --- | --- | --- | --- | --- | --- |
| *B. suillus - C. hottentotus* | 0.8704 | 1.0000 | 0.3164 | 0.3639 | **0.0383** | **0.0109** | 0.2325 | 0.2325 | 0.0750 |
| *B. suillus - F. mechowii* | 1.0000 | 0.9996 | 0.9917 | 0.9998 | 0.9999 | 0.9659 | 1.0000 | 0.9999 | 0.9859 |
| *B. suillus - F. whytei* | 1.0000 | 0.9821 | 0.1660 | 0.1660 | 0.0508 | 0.1660 | 0.5779 | 1.0000 | 0.4785 |
| *B. suillus - G. capensis* | 1.0000 | 0.9967 | 0.2609 | 0.6028 | 0.2430 | 0.4542 | 0.4303 | 1.0000 | 0.9977 |
| *B. suillus - H. argenteocinereus* | 1.0000 | 0.9414 | 0.1660 | 0.6028 | 0.6522 | 0.1660 | 0.8459 | 0.9985 | 0.9506 |
| *B. suillus - H. glaber* | 0.8936 | 0.6028 | 0.0994 | 0.0994 | 0.1187 | 0.0562 | 0.7233 | 0.9312 | 0.9506 |
| *C. hottentotus - F. mechowii* | 0.9775 | 0.9977 | 0.8789 | 0.7458 | 0.1798 | 0.2990 | 0.2260 | 0.2098 | 0.5779 |
| *C. hottentotus - F. whytei* | 0.8459 | 0.9938 | 0.9977 | 0.9954 | 1.0000 | 0.9954 | 0.9999 | 0.3192 | 0.9954 |
| *C. hottentotus - G. capensis* | 0.9775 | 0.9994 | 0.9999 | 1.0000 | 0.9994 | 0.8936 | 1.0000 | 0.5279 | 0.4303 |
| *C. hottentotus - H. argenteocinereus* | 0.8936 | 0.9721 | 0.9977 | 1.0000 | 0.9198 | 0.9954 | 0.9859 | 0.1294 | 0.7233 |
| *C. hottentotus - H. glaber* | 0.2430 | 0.7001 | 0.9859 | 0.9775 | 1.0000 | 1.0000 | 0.9977 | **0.0301** | 0.7233 |
| *F. mechowii - F. whytei* | 0.9996 | 0.9183 | 0.6566 | 0.4534 | 0.1837 | 0.7816 | 0.5206 | 1.0000 | 0.9472 |
| *F. mechowii - G. capensis* | 1.0000 | 0.9682 | 0.7816 | 0.8809 | 0.5206 | 0.9682 | 0.3892 | 0.9986 | 1.0000 |
| *F. mechowii - H. argenteocinereus* | 0.9999 | 0.8351 | 0.6566 | 0.8809 | 0.8809 | 0.7816 | 0.7816 | 1.0000 | 1.0000 |
| *F. mechowii - H. glaber* | 0.8351 | 0.4534 | 0.5206 | 0.3294 | 0.3294 | 0.5206 | 0.6566 | 0.9913 | 1.0000 |
| *F. whytei - G. capensis* | 0.9996 | 1.0000 | 1.0000 | 0.9913 | 0.9962 | 0.9986 | 1.0000 | 0.9999 | 0.8809 |
| *F. whytei - H. argenteocinereus* | 1.0000 | 1.0000 | 1.0000 | 0.9913 | 0.8809 | 1.0000 | 0.9996 | 0.9996 | 0.9824 |
| *F. whytei - H. glaber* | 0.9682 | 0.9824 | 1.0000 | 1.0000 | 0.9999 | 0.9996 | 1.0000 | 0.9682 | 0.9824 |
| *G. capensis - H. argenteocinereus* | 0.9999 | 0.9996 | 1.0000 | 1.0000 | 0.9962 | 0.9986 | 0.9962 | 0.9913 | 0.9996 |
| *G. capensis - H. glaber* | 0.8351 | 0.9472 | 0.9996 | 0.9682 | 0.9999 | 0.9682 | 0.9996 | 0.8809 | 0.9996 |
| *H. argenteocinereus - H. glaber* | 0.9472 | 0.9962 | 1.0000 | 0.9682 | 0.9682 | 0.9996 | 1.0000 | 0.9986 | 1.0000 |

**Supplement S2. Comparison of the densities of inner hair cells (IHC), outer hair cells (OHC), and the width of the OHC triad along the basilar membrane.** The F- and p-values are provided; the numbers in parentheses following species names indicate the degrees of freedom (DF).

| **Species** | **IHC** | | **OHC** | | **OHC Triad width** | |
| --- | --- | --- | --- | --- | --- | --- |
|  | F | p | F | p | F | p |
| *H. glaber* _(8, 27)_ | 9.74 | <0.0001 | 3.12 | 0.0124 | 8.47 | <0.0001 |
| *B. suillus* _(8, 45)_ | 14.83 | <0.0001 | 37.74 | 0.0001 | 9.94 | <0.0001 |
| *G. capensis* _(8, 27)_ | 7.26 | <0.0001 | 14.27 | <0.0001 | 21.83 | <0.0001 |
| *H. argenteocinereus* _(8, 27)_ | 18.62 | <0.0001 | 24.10 | <0.0001 | 25.59 | <0.0001 |
| *C. hottentotus* _(8, 45)_ | 13.25 | <0.0001 | 13.63 | <0.0001 | 13.00 | <0.0001 |
| *F. mechowii* _(8, 27)_ | 4.90 | <0.0001 | 11.39 | <0.0001 | 18.15 | <0.0001 |
| *F. whytei* _(8, 27)_ | 8.77 | <0.0001 | 5.69 | 0.0003 | 15.28 | <0.0001 |

**Results of selected pairwise comparisons of the inner hair cell (IHC) density between specific testing points along the basilar membrane (BM) length.** Tests were conducted separately for each species; * indicates statistically significant results after Bonferroni correction (α=0.05/15).

| **Tested points of BM (%)** | ***Heterocephalus***  ***glaber*** | ***Bathyergus suillus*** | ***Georychus capensis*** | ***Heliophobius argenteocinereus*** | ***Cryptomys hottentotus*** | ***Fukomys mechowii*** | ***Fukomys***  ***whytei*** |
| --- | --- | --- | --- | --- | --- | --- | --- |
| 10-20 | 0.2702 | 0.3322 | 0.3056 | 0.8594 | 0.8289 | 0.0981 | **0.0025*** |
| 20-30 | 0.4908 | 0.3258 | 0.6247 | 0.7962 | 0.7323 | 0.3269 | 0.9942 |
| 30-40 | 0.5854 | 0.3744 | 0.6511 | 0.0651 | 0.2548 | 0.2607 | 0.7551 |
| 40-50 | 0.1224 | 0.1606 | 0.0719 | 0.3050 | 0.6301 | 0.5356 | 0.5185 |
| 50-60 | 0.3859 | 0.5947 | 0.8753 | 0.0730 | 0.9211 | 0.2002 | 0.2829 |
| 60-70 | 0.2855 | 0.1446 | 0.5485 | 0.8306 | 0.0569 | 0.4631 | 0.0116 |
| 70-80 | 0.0870 | 0.1153 | 0.0338 | 0.0883 | 0.0155 | 0.9090 | 0.7331 |
| 80-90 | 0.3796 | 0.0853 | 0.9558 | 0.2377 | 0.7087 | 0.3799 | 0.6014 |
| 10-30 | 0.0732 | 0.0509 | 0.1301 | 0.6633 | 0.8997 | 0.5006 | **0.0024*** |
| 20-40 | 0.8859 | 0.0614 | 0.9705 | 0.0355 | 0.1386 | 0.0353 | 0.7496 |
| 30-50 | 0.3177 | 0.0219 | 0.1778 | 0.0041 | 0.1051 | 0.6134 | 0.7386 |
| 40-60 | 0.0159 | 0.3837 | 0.1004 | 0.0048 | 0.5615 | 0.5082 | 0.6686 |
| 50-70 | 0.0530 | 0.3540 | 0.6577 | 0.0447 | 0.0452 | 0.0439 | **0.0003*** |
| 60-80 | 0.0054 | **0.0024*** | 0.0065 | 0.0550 | **<0.0001*** | 0.3964 | 0.0290 |
| 70-90 | 0.4051 | **0.0010*** | 0.0387 | 0.0039 | 0.0052 | 0.3210 | 0.8561 |

**Results of selected pairwise comparisons of the outer hair cell (OHC) density between specific testing points along the basilar membrane (BM) length.** Tests were conducted separately for each species; * indicates statistically significant results after Bonferroni correction (α=0.05/15).

| **Tested points of BM (%)** | ***Heterocephalus***  ***glaber*** | ***Bathyergus suillus*** | ***Georychus capensis*** | ***Heliophobius argenteocinereus*** | ***Cryptomys hottentotus*** | ***Fukomys mechowii*** | ***Fukomys***  ***whytei*** |
| --- | --- | --- | --- | --- | --- | --- | --- |
| 10-20 | 0.5003 | 0.3358 | 0.0778 | 0.6141 | 0.3756 | 0.0112 | 0.7450 |
| 20-30 | 0.0327 | 0.0339 | 0.6882 | 0.2000 | 0.9442 | 0.5980 | 0.9461 |
| 30-40 | 0.4343 | 0.0848 | 0.0294 | 0.0546 | 0.0206 | 0.1651 | 0.3424 |
| 40-50 | 0.1042 | 0.9025 | 0.9705 | 0.0894 | 0.7606 | 0.3674 | 0.0542 |
| 50-60 | 0.9967 | 0.4667 | 0.1429 | 0.6289 | 0.0455 | 0.6043 | 0.9600 |
| 60-70 | 0.6534 | 0.6382 | 0.7334 | 0.6141 | 0.7236 | 0.4225 | 0.4567 |
| 70-80 | 0.3180 | 0.5767 | 0.0838 | 0.0120 | 0.4706 | 0.6138 | 0.8837 |
| 80-90 | 0.9835 | 0.8169 | 0.3708 | 0.4390 | 0.1501 | 0.0743 | 0.9634 |
| 10-30 | 0.0049 | **0.0020*** | 0.1731 | 0.0741 | 0.4145 | 0.0444 | 0.7968 |
| 20-40 | 0.0035 | **0.0001*** | 0.0756 | **0.0014*** | 0.0171 | 0.3894 | 0.3779 |
| 30-50 | 0.3992 | 0.1094 | 0.0268 | **0.0003*** | 0.0088 | 0.0221 | 0.0040 |
| 40-60 | 0.1033 | 0.3952 | 0.1331 | 0.0291 | 0.0212 | 0.1558 | 0.0482 |
| 50-70 | 0.6564 | 0.7967 | 0.0710 | 0.3234 | 0.0186 | 0.1867 | 0.4269 |
| 60-80 | 0.1477 | 0.9299 | 0.0385 | **0.0026*** | 0.2823 | 0.1913 | 0.5498 |
| 70-90 | 0.3281 | 0.4297 | 0.0087 | **0.0010*** | 0.0307 | 0.0221 | 0.8477 |

**Results of selected pairwise comparisons of the width of three rows of outer hair cell between specific testing points along the basilar membrane (BM) length.** Tests were conducted separately for each species; * indicates statistically significant results after Bonferroni correction (α=0.05/15).

| **Tested points of BM (%)** | ***Heterocephalus***  ***glaber*** | ***Bathyergus suillus*** | ***Georychus capensis*** | ***Heliophobius argenteocinereus*** | | ***Cryptomys hottentotus*** | ***Fukomys mechowii*** | ***Fukomys***  ***whytei*** |
| --- | --- | --- | --- | --- | --- | --- | --- | --- |
| 10-20 | 0.2869 | 0.0734 | 0.0909 | 0.1444 | 0.1572 | | 0.0036 | 0.0811 |
| 20-30 | 0.2486 | 0.0181 | 0.1885 | 0.1006 | 0.1116 | | 0.0524 | 0.2669 |
| 30-40 | 0.5944 | 0.5193 | 0.0388 | 0.0177 | 0.0770 | | 0.1458 | 0.3413 |
| 40-50 | 0.4777 | 0.4309 | 0.7071 | 0.1444 | 0.8597 | | 0.1960 | 0.6342 |
| 50-60 | 0.7226 | 0.4740 | 0.0603 | 0.8552 | 0.3767 | | 0.8716 | 0.0811 |
| 60-70 | 0.3749 | 0.7746 | 0.5730 | 0.1444 | 0.0770 | | 0.1960 | 0.5258 |
| 70-80 | 0.1102 | 0.5668 | 0.0603 | 0.0447 | 0.1572 | | 0.4190 | 0.0811 |
| 80-90 | 0.4777 | 0.2520 | 0.1329 | 0.5841 | 0.0770 | | 0.8716 | 0.2669 |
| 10-30 | 0.0265 | **<0.0001*** | **0.0026*** | **0.0019*** | **0.0026*** | | **<0.0001*** | 0.0043 |
| 20-40 | 0.0918 | **0.0026*** | **0.0007*** | **0.0001*** | **0.0008*** | | **0.0007*** | 0.0392 |
| 30-50 | 0.2141 | 0.1521 | 0.0146 | **0.0001*** | 0.1116 | | 0.0060 | 0.1535 |
| 40-60 | 0.2869 | 0.1327 | 0.0242 | 0.2015 | 0.4794 | | 0.1458 | 0.0264 |
| 50-70 | 0.2141 | 0.6675 | 0.1885 | 0.2015 | 0.0080 | | 0.1458 | 0.0174 |
| 60-80 | 0.0130 | 0.3902 | 0.1885 | **0.0005*** | 0.7236 | | 0.6278 | 0.0174 |
| 70-90 | 0.3749 | 0.5668 | **0.0007*** | 0.1444 | 0.7236 | | 0.5179 | 0.5258 |
